# The diagnostic value and clinical relevance of high frequency ultrasound and shear wave elastography in systemic sclerosis: an observational monocentric study

**DOI:** 10.1007/s10067-024-07145-6

**Published:** 2024-10-05

**Authors:** Shengnan Yu, Haiting Peng, Xiaoyun Yang, Sha Ma, Juan Yu, Dachen Zuo, Fayou Li, Juan Wang, Yonghong Yang, Zijing Yin, Weiqing Zhao, Jing Wang

**Affiliations:** 1 Department of Rheumatology and Immunology, The First People’s Hospital of Yunnan Province, The affiliated hospital of Kunming University of Science and Technology, Jinbi Road, No.157, Kunming, 650032 Yunnan Province China; 2 Department of Rheumatology and Immunology, The Guigang People’s Hospital, The Eighth Affiliated Hospital of Guangxi Medical University, Zhongshan Middle Road, No.1, Guigang, 537100 Guangxi Province China; 3 Department of Ultrasound, The First People’s Hospital of Yunnan Province, The affiliated hospital of Kunming University of Science and Technology, Jinbi Road, No.157, Kunming, 650032 Yunnan Province China

**Keywords:** Clinical relevance, Diagnosis, High frequency ultrasound, Shear wave elastography, Systemic sclerosis

## Abstract

To unravel the features of skin involvement in patients with systemic sclerosis (SSc) by high frequency ultrasound (HFU) and shear wave elastography (SWE). To assess the ultrasound capabilities to distinguish SSc patients from healthy controls (HCs). We recruited a cohort of SSc patients in this cross-sectional study. HFU and SWE were used to quantify skin thickness and skin stiffness. Receiver operating characteristic (ROC) curve analysis was used to assess the diagnostic capabilities of ultrasound in SSc. The correlation analysis was used to evaluate the clinical relevance of ultrasound measurements in SSc. 20 consecutive SSc patients and 20 age-, gender- and body mass index-matched HCs were included. The skin thickness and stiffness were significantly greater in SSc patients compared with HCs. Patients with high disease activity had higher skin thickness and stiffness compared with patients with low disease activity. The area under the ROC curve (AUC) of the dorsum of middle fingers assessed by HFU was 0.847 (95% CI, 0.761–0.933). The AUC of the forearms and dorsum of hands assessed by SWE were 0.909 (95% CI, 0.829–0.989) and 0.879 (95% CI, 0.807–0.951). Further, the combined HFU and SWE tests displayed the best diagnostic performance with an AUC of 0.980 (95% CI, 0.939–1.000). A significant positive correlation between the ultrasound measurements and the modified Rodnan skin score (mRSS) was observed. The application of ultrasound can assist with disease diagnosis, it is necessary to develop a standard operating protocol to help with future implementation of ultrasound in SSc.

**Supplementary Information:**

The online version contains supplementary material available at 10.1007/s10067-024-07145-6.

**Table:** 

**Key Points** *• The application of HFU and SWE can assist with SSc diagnosis.* *• The skin thickness and stiffness measured by HFU and SWE positively correlate with mRSS.* *• HFU and SWE enable quantitative measurement of skin thickness and stiffness of different skin sites.*		

## Introduction

Systemic sclerosis (SSc) is an autoimmune connective tissue disease of unknown etiology, characterized by fibrosis of the skin and internal organs and vasculopathy [1, 2]. In patients with early SSc, which usually refers to those within the first 3 to 5 years of the onset of symptoms, skin thickening and hardening has the greatest impact on quality of life, causing pain, intractable itching, decreased mobility and functional limitation [3]. Cohort studies and clinical trials show that the extent of skin involvement is associated with disease survival, progression, and mortality rate [4–6], as well as organ involvement such as pulmonary, cardiovascular and renal progression [6, 7], while improvement in the skin condition is associated with better prognosis [8]. Therefore, reliable testing and monitoring of skin involvement is necessary in the early diagnosis and holistic management of SSc.

The modified Rodnan skin score (mRSS) is one of the main measures currently used for the semi-quantitative assessment of SSc skin involvement [9, 10]. The mRSS is assessed by palpation at 17 skin sites and scored on a 0 to 3 scale, giving a total score of 0 to 51 [3]. While the mRSS is valuable in the outpatient setting due to its simplicity and convenience, it is important to acknowledge its limitations and challenges, such as significant inter-observer variability, relatively poor accuracy, low sensitivity to subtle changes, and inability to differentiate later-stage disease due to tethered skin [11, 12]. Thus, other outcome measures of skin involvement, including non-invasive imaging methods, are attracting increasing interest for application in SSc.

High frequency ultrasound (HFU), or the ultrasound using a frequency of at least 10 megahertz (MHz), is one of the main non-invasive imaging methods used for real-time skin imaging [13, 14]. Compared to conventional ultrasound, HFU has further enhanced imaging clarity and can precisely measure thickness of different skin layers [13, 15]. In addition, shear wave elastography (SWE) mode uses an acoustic radiation force pulse sequence to generate shear waves, which by itself provides a local stress and generates local displacement in the tissue, allowing both qualitative and quantitative measurement of tissue elasticity [16, 17]. The distribution of shear wave velocities (SWV) at each pixel is directly related to the shear modulus, an absolute measure of the tissue’s elastic properties. In general, shear waves propagate faster through stiffer contracted tissue, and the long axis of tendon and muscle [16]. With HFU and elastography becoming available in a clinical setting, some studies have reported good repeatability within and across examiners as well as higher sensitivity in the ultrasound evaluation of skin thickness and stiffness [18, 19], indicating that the ultrasonographic measurement a more objective, sensitive and reproducible tool. However, additional studies will be needed to assess the validity and clinical significance of the ultrasonographic measurement in SSc.

The aim of this study were (1) to investigate the diagnostic values of HFU and SWE at different skin sites, and (2) to evaluate the clinical correlations of the ultrasound assessment in SSc.

## Methods

### Study population

We consecutively recruited a cohort of SSc patients and healthy controls (HCs) matched by age, gender, and body mass index (BMI) from the First People’s Hospital of Yunnan Province between January 2022 and January 2023. All patients met the American College of Rheumatology (ACR) / European League Against Rheumatism (EULAR) 2013 criteria for SSc [20]. Patients overlapped with other connective tissue diseases, or demonstrated signs of skin thickening, inflammation or scaling due to other skin diseases, and those receiving radiochemotherapy were excluded. Clinical profiles of patients were recorded at enrollment. mRSS and HFU were used for the evaluation of skin thickening. SWE was used to measure skin stiffness. European Scleroderma Trial and Research group Disease Activity Index (EUSTAR-DAI) was used to assess the disease activity. The study was approved by the The Medical Ethics Committee of the First People’s Hospital of Yunnan Province. All patients gave their informed consent at the time of assessment.

### Clinical assessments and data collection

The clinical data of each eligible participant were collected at the enrollment. The collected data included (1) demographics: sex, age, BMI; (2) clinical features and auxiliary examination of SSc: disease duration, mRSS, Raynaud’s phenomenon (RP), digital ulcers (DU), interstitial lung disease (ILD), synovitis, erythrocyte sedimentation rate (ESR), C-reactive protein (CRP), disease activity index (EUSTAR-DAI), anti-Scl-70.

The mRSS is assessed in 17 different skin areas by palpation. The scoring of each individual cutaneous area is scored on a 0–3 scale. mRSS = 0 is defined as “normal skin” where the examiner appreciates fine wrinkles but no skin thickening. mRSS = 1 is defined as definite but “mild” skin thickening where the examiner can easily make skin folds between 2 fingers; fine wrinkles are acceptable. mRSS = 2 is defined as “moderate” skin thickness with difficulty in making skin folds and no wrinkles. mRSS = 3 is defined as “severe” skin thickness with inability to make skin folds between 2 examining fingers [10, 21].

The level of disease activity was calculated according to the EUSTAR 2016 revised standard for disease activity of SSc patients [22]. EUSTAR-DAI is a weighted 10-point disease activity index including 6 items: Δ-skin = 1.5 (Δ means skin worsening as evaluated by the patient during the month before enrollment), mRSS > 18 = 1.5, digital ulcers = 1.5, tendon friction rubs = 2.25, CRP > 1 mg/dL = 2.25 and diffusing capacity of the lung for CO (DLCO) % predicted < 70% = 1.0. EUSTAR-DAI ≥ 2.5 is defined as active disease.

### Skin ultrasound examination

Aplio i900 ultrasound system (Canon, Japan) equipped with a 24-MHz linear probe was used to evaluate skin thickness and stiffness. HFU and SWE measurements were made on all or part of the following 17 skin sites [23]: dorsum of middle fingers (proximal interphalangeal joint), dorsum of hands (the metacarpal interspace of index and middle fingers, 2 cm proximal to the metacarpophalangeal joints), anterior forearms (10 cm proximal to styloid process of ulna), anterior upper arms (10 cm proximal to medial epicondyle), forehead, anterior chest wall (between jugular notch and sternal angle), anterior abdominal wall (10 cm below xiphoid), legs (10 cm proximal to patella), lateral lower legs (10 cm proximal to lateral malleolus), dorsum of feet (2 cm proximal to the 1^st^ and 2^nd^ metatarsophalangeal joints) (Supplementary Figure S1).

**Fig. 1:**
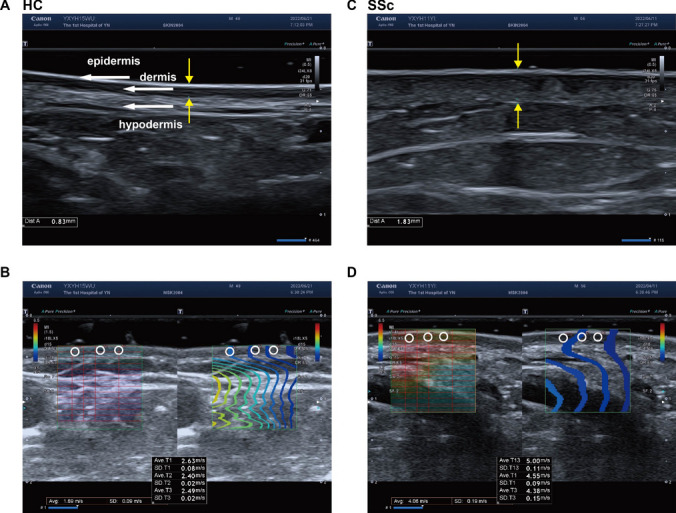
Representative HFU and SWE images. HFU highlights the epidermis, dermis and hypodermis (white arrows) at the forearm (**A**, **C**). The yellow arrows indicate the thickness of skin. The ROI (colored squares) was superimposed on the SWE image (**B**, **D**). The elastic modulus values of the dermis layer were measured inside the 1-mm-diameter white small circle named Q-box. **A**, **B** are representative images of healthy controls; **C**, **D** are representative images of SSc patients

To quantify skin thickness, the probe was maintained perpendicular to the skin surface without pressure during the assessment process. Skin thickness measurement includes the epidermis and dermis (Fig. 1). To quantify skin stiffness, SWE mode was activated and a square area on the target skin site was defined as the region of interest (ROI). A color-coded elastogram was superimposed on the SWE image as ROI. The image was saved when stable color was obtained, then a 1-mm diameter Q-box (Fig. 1) was drawn within dermis. SWV was then automatically calculated in meters per second (m/s) by the ultrasound system, higher SWV reflected higher tissue stiffness [24, 25]. All measurements were made by experienced operators in triplicate and averaged.

### Statistical analysis

Statistical analyses were conducted using SPSS V.23.0. Graphpad Prism 9 and Origin 2022 software were used to produce graphs. Patient characteristics were presented as mean ± standard deviation (SD) or median and interquartile range (IQR) depending on the level of resemblance to the normal distribution. Comparisons between HCs and SSc patients were analyzed by Student’s t-tests for normally distributed continuous variables, or Mann–Whitney U tests for skewed continuous variables. Receiver operating characteristic (ROC) curve analysis was used to predict the sensitivity and specificity of skin thickness detection by HFU or skin stiffness detection by SWE. Area under the ROC curve (AUC) was calculated and optimal cutoff values were obtained by maximizing the Youden index from the estimated curves. To create an ROC curve for combined tests, we first estimated a logistic regression model with the combined tests as the explanatory variables, and saved the predicted probabilities from the model. Then we used the predicted probability as the "test" variable in the ROC procedure. For any diagnostic techniques to be acceptable, the AUC must be greater than 0.8. In addition, when comparing the performance of two or more diagnostic tests, the ROC curve with the largest AUC is considered to have a better diagnostic performance [26]. The Spearman correlation analysis was used to test correlation. P values ≤ 0.05 were considered statistically significant. Bonferroni correction for the multiple correlations between HFU/SWE and clinical parameters were applied, adjusted P values ≤ 0.05 (1/n) were considered statistically significant.

## Results

### Patient characteristics

In total, 20 SSc patients were consecutively recruited and 20 HCs matched by age, gender, and BMI were included in our study. The baseline characteristics of the study population were presented in Table 1. Among 20 SSc patients, 65% were female and the average mRSS was 18.90 (7.28). Most of the patients had Raynaud’s phenomenon (95%) and interstitial lung disease (75%). The average CRP and ESR levels were 7.80 (8.57) mg/L and 29.25 (21.17) mm, respectively. The mean disease activity index was 3.28 (1.50), among which 12 patients had high disease activity (EUSTAR-DAI ≥ 2.5), 8 presented with low disease activity (EUSTAR-DAI < 2.5). For the baseline antibody profile of the patients, 40% (8/20) were positive for anti-Scl-70 antibody.

**Table 1:** Demographics and clinical characteristics of the study populations

Characteristics of patients	Whole cohort (*n* = 20)
Basic characteristics	
Female, % (*n*)	65.0% (13/20)
Age, mean, (years)	49.35 ± 8.34
BMI, mean, (kg/m^2^)	22.97 ± 4.08
Disease duration, median, (years)	3.00 (1.00–5.00)
mRSS, mean	18.90 ± 7.28
RP, % (*n*)	95% (19/20)
DU, % (*n*)	25% (5/20)
ILD, % (*n*)	75% (15/20)
Synovitis, % (*n*)	20% (4/20)
Antibody profiles	
anti-Scl-70, % (*n*)	40% (8/20)
Baseline disease activity measures	
CRP, mean, mg/L	7.80 ± 8.57
ESR, mean, mm	29.25 ± 21.17
EUSTAR-DAI, mean	3.28 ± 1.50

Values are presented as mean (SD) or median (IQR), as applicable

There were 20 people in the HC group, in which 65.0% were females (13/20). The average age was 48.60 ± 8.85 years old. The average BMI was 21.93 ± 3.34 kg/m^2^. The mean mRSS was 3.30 ± 1.13

*CRP*, C-reactive protein; *ESR*, erythrocyte sedimentation rate

### Skin thickness and stiffness measured by ultrasound

**Fig. 2:**
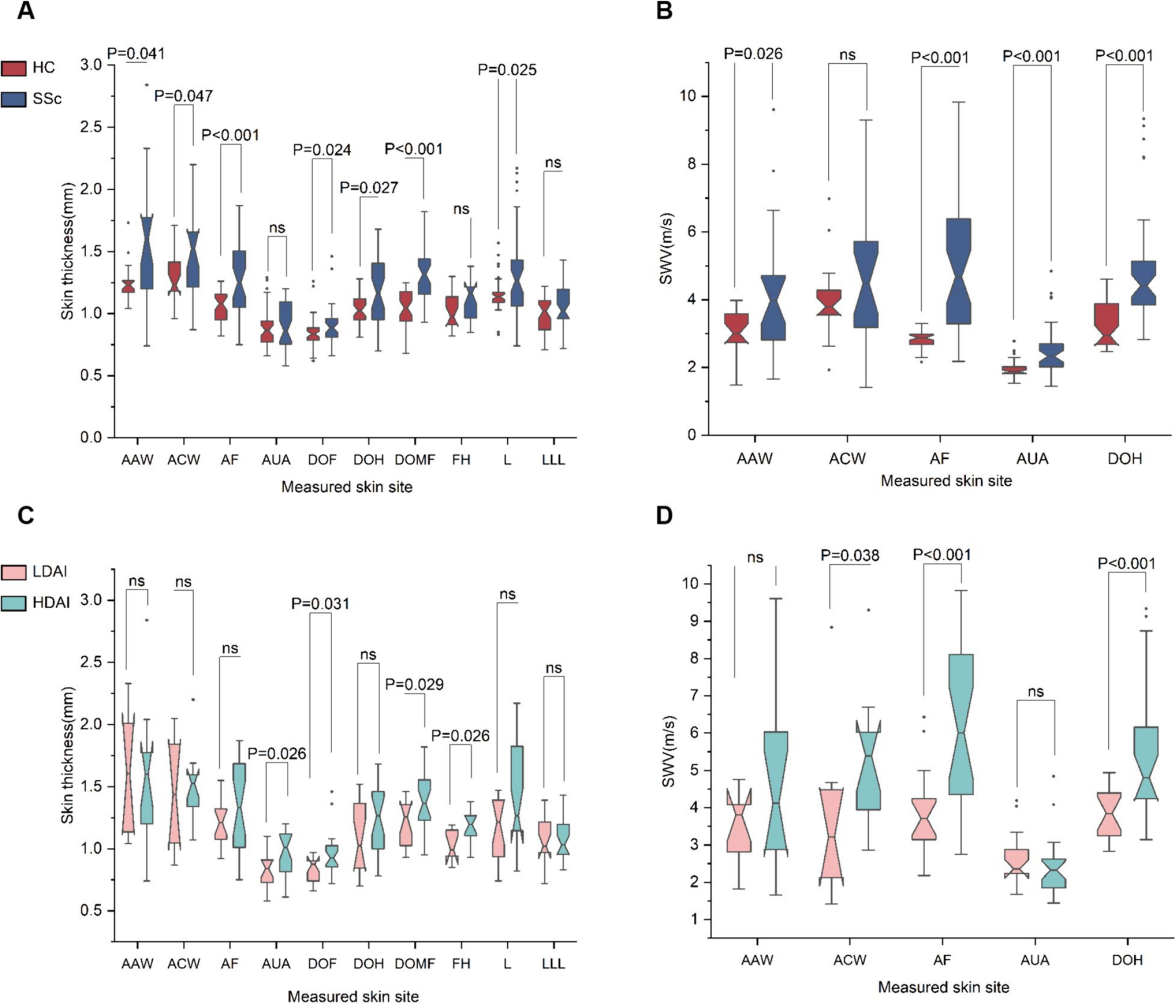
Skin thickness and stiffness measured by ultrasound. **A** Comparison of the skin thickness evaluated by HFU in HCs and SSc patients. **B** Comparison of skin stiffness evaluated by SWE in HCs and SSc patients. **C** Comparison of the skin thickness evaluated by HFU in low and high disease activity SSc patients. **D** Comparison of the skin stiffness evaluated by SWE in low and high disease activity SSc patients. Notched boxes represent the 25th and 75th percentile of measures. The median (line within the box), maximum and minimum values (top and bottom lines) are shown. P values were determined by the Mann–Whitney U test. Abbreviations: AAW, anterior abdominal wall; ACW, anterior chest wall; AF, anterior forearms; AUA, anterior upper arms; DOF, dorsum of feet; DOH, dorsum of hands; DOMF, dorsum of middle fingers; FH, forehead; HDAI, high disease activity; L, legs; LDAI, low disease activity; LLL, lateral lower legs

We compared the skin thickness in SSc patients and HCs. Of 17 skin sites measured by HFU, the skin thickness of the dorsum of middle fingers, dorsum of hands, forearms, chest wall, abdominal wall, legs, and dorsum of feet were significantly higher in SSc patients than in HCs, no statistically significant differences were found for the upper arms (*P* = 0.532), forehead (*P* = 0.084) and lower legs (*P* = 0.052) (Fig. 2A and Supplementary Table S1). For the evaluation of skin stiffness measured by SWE, we only performed the test on 8 skin sites, mainly because some areas such as fingers and phalanxes lack sufficient soft tissue. We found that the skin stiffness values of the middle fingers, forearms, upper arms and abdominal wall in patients with SSc were significantly greater than those of the controls, no statistically significant difference was found for the anterior chest wall (Fig. 2B and Supplementary Table S1).

Further, we divided the patients into two subgroups based on disease activity index. A EUSTAR-DAI greater than or equal to 2.5 was classified as high disease activity (HDAI-SSc), and a EUSTAR-DAI of less than 2.5 was defined as low disease activity (LDAI-SSc). We found that the skin thickness of fingers, upper arms, forehead and feet were significantly higher in HDAI-SSc than in LDAI-SSc group (Fig. 2C and Supplementary Table S2). Similarly, the skin stiffness values of the hands, forearms, and abdominal wall were markedly increased in HDAI-SSc than in LDAI-SSc group (Fig. 2D and Supplementary Table S2). Collectively, these results suggested that the skin thickness and stiffness vary in different skin areas in SSc patients, moreover, patients with active disease presented with more severe skin involvement.

### Diagnostic capabilities of HFU and SWE in SSc

**Fig. 3:**
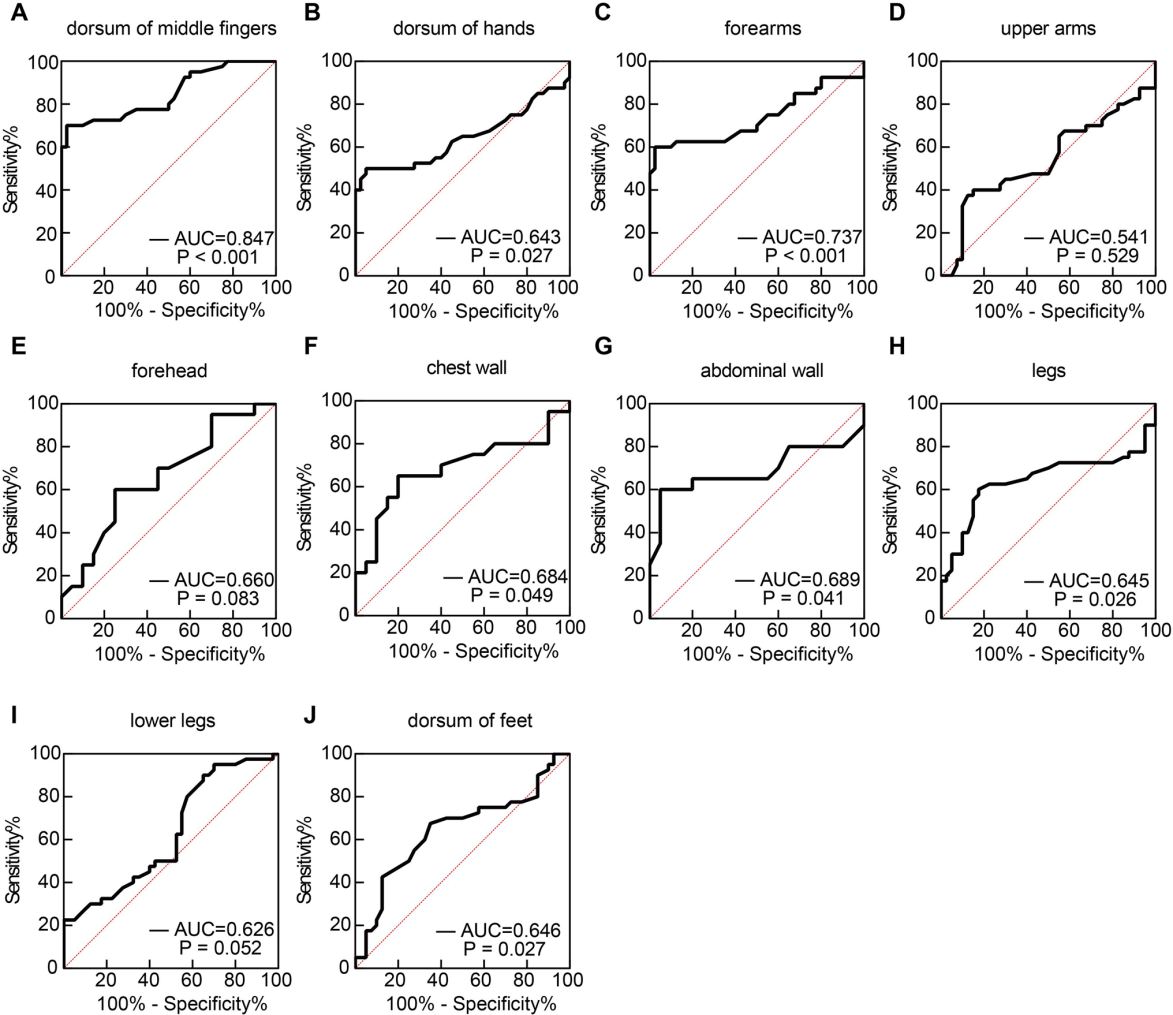
ROC curves for evaluation of HFU as a potential diagnostic tool for SSc at different skin sites. Skin thickness measured by HFU was evaluated for prediction of SSc, resulting in an AUC shown at the lower right corner of each ROC curve

**Fig. 4:**
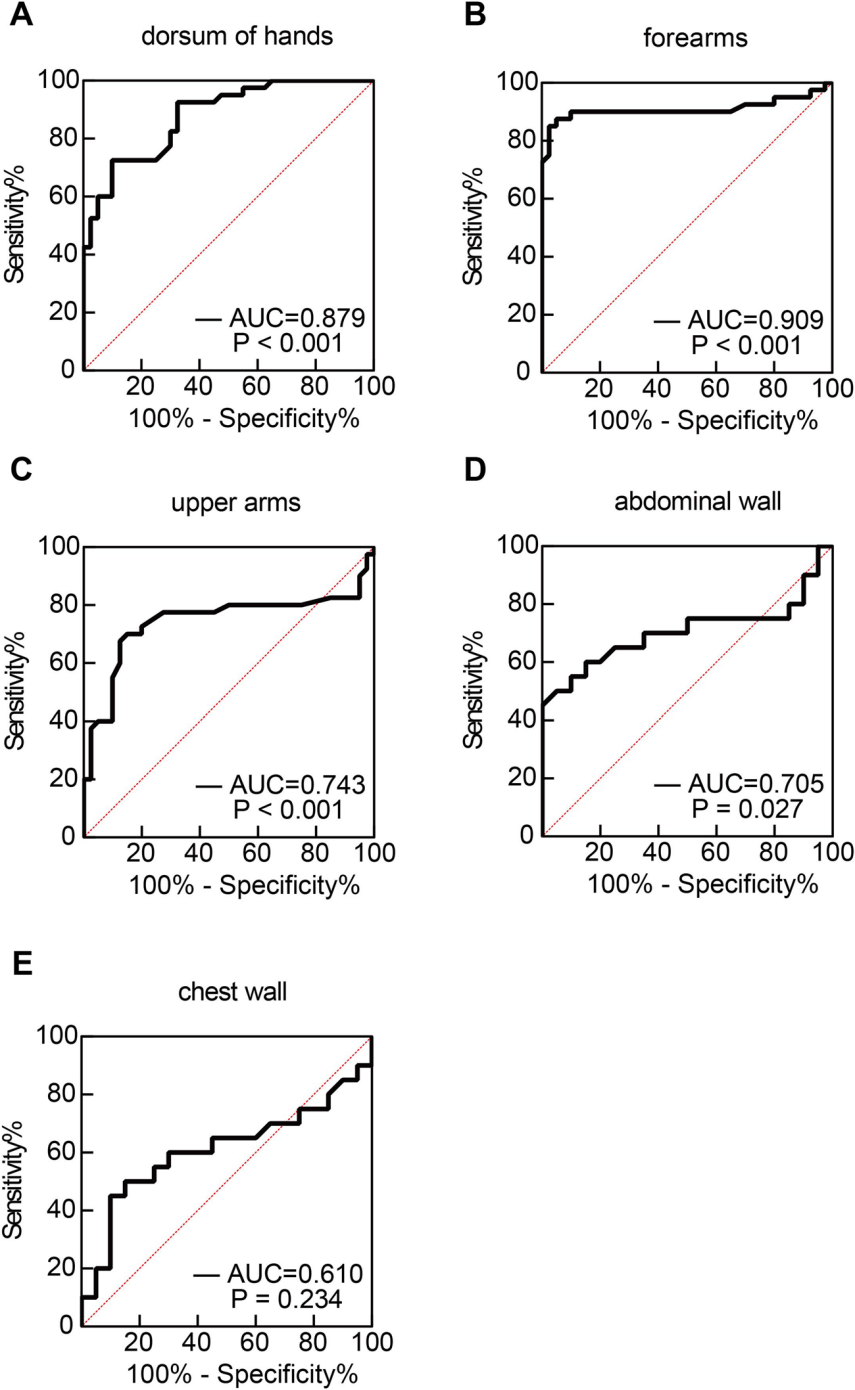
ROC curves for evaluation of SWE as a potential diagnostic tool for SSc at different skin sites. Skin stiffness measured by SWE was evaluated for prediction of SSc, resulting in an AUC shown at the lower right corner of each ROC curve

In order to investigate the diagnostic performance of HFU in differentiating skin thickening between SSc patients and HCs, we calculated the sensitivity and specificity for HFU of 17 skin sites by ROC curve analysis. The AUC value of the dorsum of middle fingers was the highest (0.847, 95% CI, 0.761–0.933), indicating a good discriminatory ability and an acceptable diagnostic performance. The sensitivity and specificity were 70.00% and 97.50%, respectively. The cutoff value for evaluation of the skin at fingers was 1.220 mm. The AUC of the forearms was 0.737 (95% CI, 0.621–0.853), which means having a fair discriminative capability (Fig. 3 and Supplementary Table S3). We also studied the diagnostic performance of SWE in evaluating SSc skin stiffness in patients. We calculated the sensitivity and specificity for the SWE of 8 skin sites by ROC curve analysis. The AUC value of the forearms was the highest (0.909, 95% CI, 0.829–0.989), indicating an excellent discriminatory ability. The sensitivity and specificity were 87.50% and 95.00%, respectively. The cutoff value for evaluation of forearm skin was 3.125 m/s. The AUC value of the dorsum of hands was 0.879 (95% CI, 0.807–0.951), indicating a good discriminatory ability. The sensitivity and specificity were 72.50% and 90.00%, respectively. The cutoff value for evaluation of the skin at hands was 3.960 m/s. Thus, SWE of the forearms and the dorsum of hands were considered acceptable diagnostic tests. The AUC values of the upper arms (0.743, 95% CI, 0.624–0.863) and anterior abdominal wall (0.705, 95% CI, 0.530–0.880) were greater than 0.7, which means having fair discriminative capabilities (Fig. 4 and Supplementary Table S4).

**Fig. 5:**
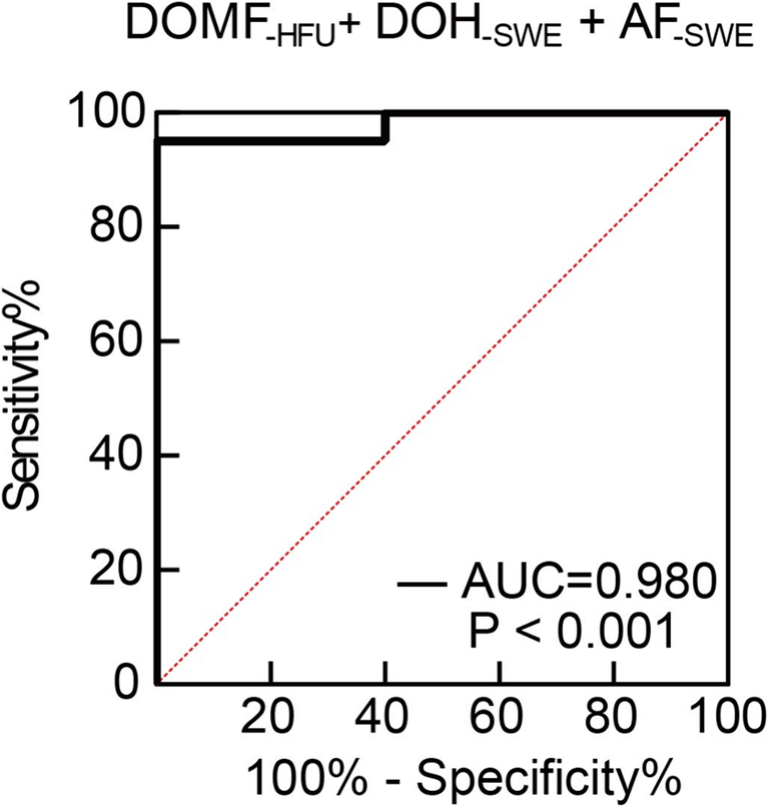
ROC curve for evaluation of combined diagnostic tests as a potential diagnostic tool for SSc

Diagnostic accuracy can be improved considerably by combining multiple markers [27], so we selected 3 diagnostic tests, namely skin thickness of the dorsum of middle fingers tested by HFU, skin stiffness of the dorsum of hands and anterior forearms tested by SWE, with good or excellent discriminatory abilities to create a new ROC curve. The AUC of the combined tests was 0.980 (95% CI, 0.939–1.000). The sensitivity and specificity were 95.00% and 100.00%, respectively (Fig. 5). Comparing to individual ultrasound test, the combination of HFU and SWE dramatically increased the diagnostic accuracy. Collectively, the ROC analysis indicated that HFU test of fingers, SWE test of forearms or hands can well identify SSc patients, combining HFU and SWE measurements can improve diagnostic accuracy.

### The clinical significance of HFU and SWE in SSc

**Fig. 6:**
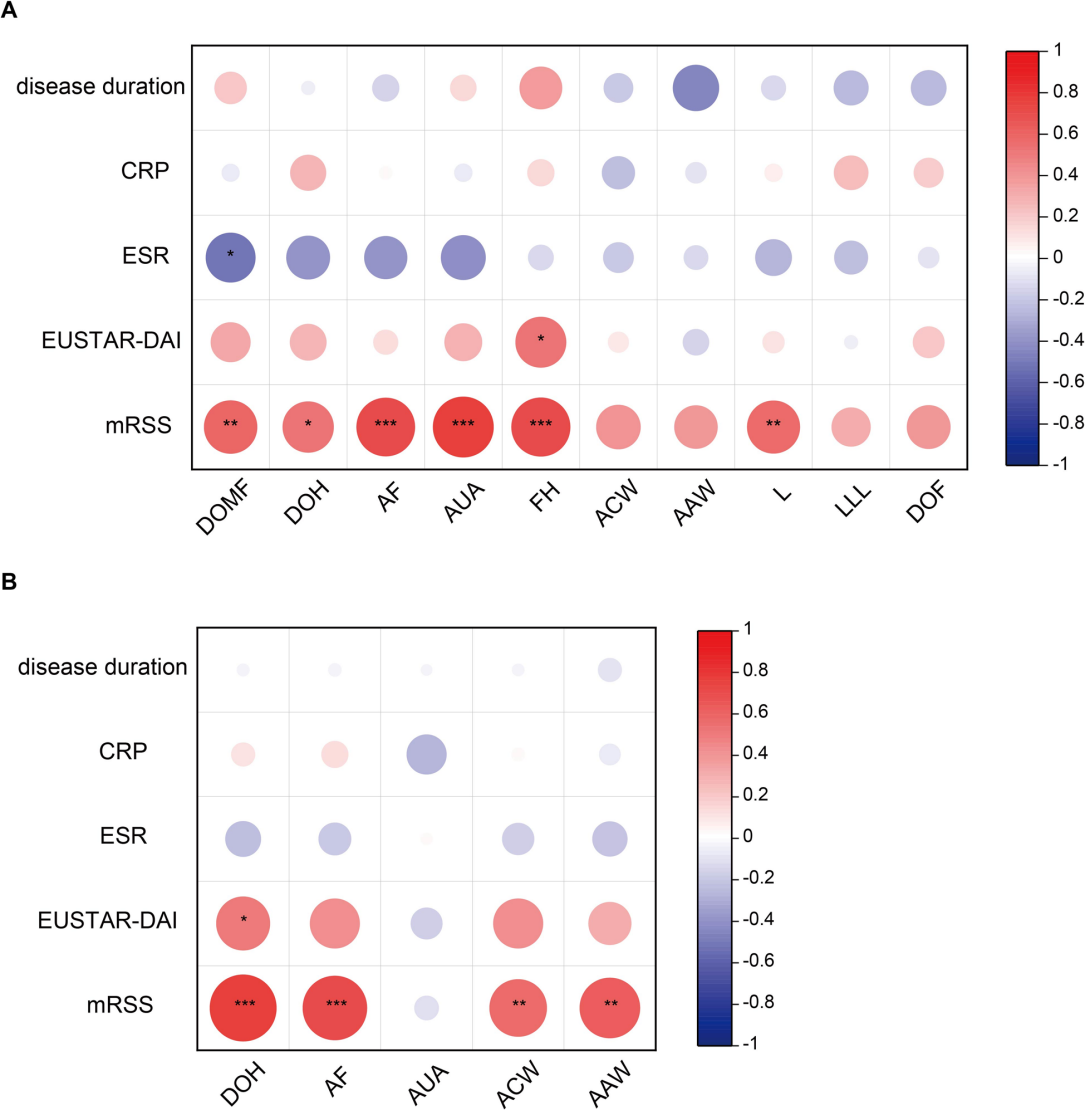
Correlations of HFU and SWE measures with clinical parameters. **A** The correlation between HFU measures and clinical parameters. **B** The correlation between SWE measures and clinical parameters. The red circle indicates positive correlation, and the blue circle indicates negative correlation. The darker the color, the larger the diameter, the stronger the correlation. *P* values and correlation coefficients were determined by the Spearman correlation analysis. *p ≤ 0.05, **p ≤ 0.01, ***p ≤ 0.001

In order to understand the clinical significance of HFU and SWE in assessing SSc patients’ skin involvement, we analyzed the correlations of HFU and SWE measures with 5 clinical parameters, including disease duration, CRP, ESR, EUSTAR-DAI and mRSS. We found that the skin thickness of the forehead measured by HFU was significantly positively correlated with EUSTAR-DAI (*r* = 0.606, *P* = 0.005). The skin thickness of the dorsum of middle fingers (*r* = 0.549, *P* = 0.010), dorsum of hands (*r* = 0.537, *P* = 0.015), forearms (*r* = 0.692, *P* = 0.001), upper arms (*r* = 0.675, *P* = 0.001), forehead (*r* = 0.786, *P* = 0.0001) and legs (*r* = 0.572, *P* = 0.008) were significantly positively correlated with mRSS (Fig. 6A). In the meantime, the skin stiffness of the dorsum of hands measured by SWE was significantly positively correlated with EUSTAR-DAI (*r* = 0.502, *P* = 0.024). The skin stiffness of the dorsum of hands (*r* = 0.767, *P* = 0.0001), forearms (*r* = 0.718, *P* = 0.0004), anterior chest wall (*r* = 0.568, *P* = 0.009) and anterior abdominal wall (*r* = 0.631, *P* = 0.003) were significantly positively correlated with mRSS. There were no significant correlations between ultrasound measures with disease duration, CRP and ESR levels (Fig. 6B).

Moreover, we made Bonferroni correction in the correlation tests (Bonferroni correction P of HFU was 0.001 (0.05/50), Bonferroni correction P of SWE was 0.002 (0.05/25). The skin thickness of the forearms (*r* = 0.692, *P* = 0.001), upper arms (*r* = 0.675, *P* = 0.001), and forehead (*r* = 0.786, *P* = 0.0001) were significantly positively correlated with mRSS. The skin stiffness of the dorsum of hands (*r* = 0.767, *P* = 0.0001), forearms (*r* = 0.718, *P* = 0.0004) were significantly positively correlated with mRSS. Other correlations were no longer significant after Bonferroni correction.

### Discussion

The skin involvement is an important topic in SSc, not only because the skin is the most visible, characteristic and accessible ‘window’ into SSc disease process, but also because the skin thickening and hardening substantially compromises the quality of life in patients. The current study applied HFU and SWE tests in detecting SSc skin involvement, and verified the diagnostic efficiency and clinical significance of the use of ultrasound in SSc. Our data supported the use of skin ultrasound for examining skin involvement of SSc patients in clinical practice.

In our study, we found that HFU/SWE determined skin thickness and stiffness of the fingers, hands, forearms, and feet in SSc patients were significantly greater than HCs, which is consistent with the characteristics of skin involvement in the limbs of SSc [1, 3]. Similar to our findings, Nerado [28] et al. found that the skin of fingers was significantly thicker in SSc patients than in controls. Li [29] et al. showed that the skin thickness of hands, forearms and phalanx were markedly higher than that of HCs. Yang [25] et al. compared the skin elastic modulus values measured by SWE, and found the SWVs were significantly higher in patients at middle fingers, forearms, anterior chest wall and abdomen compared with those of HCs at the corresponding sites. Aryan [17] et al. however, considered the finger elastography infeasible because of the anatomical structures of this area lack soft tissues, the presence of a bone in the field of elastography may affect SWE testing. To be noted, our study discovered a mismatch between HFU and SWE test results, for instance, the skin stiffness of the upper arms was markedly greater than that of HCs, whereas the skin thickness of upper arms in SSc patients was not significantly thicker than in controls. These results suggested a selective HFU or SWE examination of the skin sites in SSc patients may improve the detection of the disease, clinicians should choose suitable ultrasound test in different skin regions to obtain reliable test results.

Further, we discussed the diagnostic capabilities of HFU and SWE in SSc by ROC curve analysis, and found that the diagnostic performances differ greatly in different skin areas. In a study conducted by Nerado [28] et al. the AUC corresponding to dermis and hypodermis layers’ thickness in fingers measured by HFU displayed the best discrimination. Yang [25, 30] et al. indicated that the accuracy of SWE measurements was excellent for the bilateral fingers (AUC = 0.974 and 0.949, respectively), good for the left forearm (AUC = 0.841), and low for chest wall in SSc patients. Sobolewski [31] et al. claimed that SWE was highly accurate for distinguishing the skin stiffness of the fingers’ skin evaluated in SSc patients (AUC = 0.961, sensitivity 0.897–0.923, specificity 0.929–0.964). Our data and the above analysis revealed that the sensitivity, specificity, and diagnostic accuracy of the two ultrasound methods were site dependent, and also reminded us that the combination of HFU and SWE may help achieve higher diagnostic accuracy.

Apart from studying the diagnostic capabilities, we further found that the mRSS was significantly positively correlated with the skin thickness and stiffness over several skin sites, suggesting ultrasound can be used as an alternative tool for quantitative skin involvement assessment. However, EUSTAR-DAI only showed positive correlations with the skin thickness of forehead, and skin stiffness of hands. We also analyzed the correlations between disease duration, inflammatory factors (e.g. ESR, CRP) with ultrasound measures, but did not manage to find significant correlations. We made Bonferroni correction after calculating multiple correlations, some of the significant correlations no longer existed. A possible explanation was that the Bonferroni correction excessively reduced statistical power for it is too conservative. Similar to our study results, some previous studies claimed that mRSS was positively correlated with the skin stiffness and thickness assessed by ultrasound [23, 32]. Another study conducted by Li [29] et al. however, reported different correlations, they found a positive relationship between the HFU measurements and disease activity parameters (EUSTAR-DAI, CRP), and a negative correlation between disease duration and skin thickness in SSc patients. The skin of SSc patients is proved to be thicker in the edematous phase than in the later fibrotic phase [18], some of the enrolled patients in our study had a disease course of more than 5 to 10 years, the high heterogeneity of SSc disease progression and small sample size of our study may explain why we found insufficient relations between ultrasound measurements with disease activity.

The limitations of this study include: First, the findings of a single-center study may limit the external generalisability. Second, the sample size was relatively small, we were not able to further stratify enough patients into subgroups, thus failed to discuss topics such as the correlations between internal organ involvement with skin ultrasound measurements. Third, we did not perform inter-observer variability assessment and compare the inter-observer variability of mRSS and ultrasound, which may lead to bias of the results. Nevertheless, the current study indeed added important information to our understanding of ultrasound usage in SSc, and proved the validity and clinical significance of ultrasound in the assessment of SSc skin involvement. We hope this study can assist with future adoption of ultrasound as a validated diagnostic tool and an outcome measure in either later-phase multi-center studies or clinical practice.

## Conclusions

As a non-invasive imaging technology, our study proved that comparing with semi-quantitative measurement mRSS, the application of HFU and SWE can assist with disease diagnosis, thus allows for more timely intervention and optimal prognosis management. However, the current skin ultrasound use in SSc patients still lacks a standard operating procedure, it is necessary to develop a protocol to help with future implementation of ultrasound in SSc.

## Supplementary Information

Below is the link to the electronic supplementary material.

Supplementary file1 (DOCX 179 KB) Schema of 17 skin sites for skin ultrasound examination. (1-2) dorsum of middle fingers (proximal interphalangeal joint). (3-4) dorsum of hands (the metacarpal interspace of index and middle fingers, 2 cm proximal to the metacarpophalangeal joints). (5-6) anterior forearms (10 cm proximal to styloid process of ulna) (7-8) anterior upper arms (10 cm proximal to medial epicondyle). (9) forehead. (10) anterior chest wall (between jugular notch and sternal angle). (11) anterior abdominal wall (10 cm below xiphoid). (12-13) legs (10 cm proximal to patella). (14-15) lateral lower legs (10 cm proximal to lateral malleolus). (16-17) dorsum of feet (2 cm proximal to the 1st and 2nd metatarsophalangeal joints)

## Data Availability

All data are available in the main text or the supplementary materials, or available on request from the authors.
