# Supplementary material for: The diagnostic value and clinical relevance of high frequency ultrasound and shear wave elastography in systemic sclerosis: an observational monocentric study
Source: Clin Rheumatol. 2024 Oct 5;43(12):3759–69. doi: 10.1007/s10067-024-07145-6 (PMC11582183; doi:10.1007/s10067-024-07145-6)
Supplement: Supplementary file 1 — Supplementary file1 (DOCX 179 KB) Schema of 17 skin sites for skin ultrasound examination. (1-2) dorsum of middle fingers (proximal interphalangeal joint). (3-4) dorsum of hands (the metacarpal interspace of index and middle fingers, 2 cm proximal to the metacarpophalangeal joints). (5-6) anterior forearms (10 cm proximal to styloid process of ulna) (7-8) anterior upper arms (10 cm proximal to medial epicondyle). (9) forehead. (10) anterior chest wall (between jugular notch and sternal angle). (11) anterior abdominal wall (10 cm below xiphoid). (12-13) legs (10 cm proximal to patella). (14-15) lateral lower legs (10 cm proximal to lateral malleolus). (16-17) dorsum of feet (2 cm proximal to the 1st and 2nd metatarsophalangeal joints) [file 10067_2024_7145_MOESM1_ESM.docx]

**Supplementary Table S1** Comparison of skin thickness and stiffness between SSc group and control group

| **Measured skin sites** | **Parameters** | | |
| --- | --- | --- | --- |
| **Skin thickness** | **SSc (mm)** | **HC (mm)** | **P value** |
| Dorsum of middle fingers | 1.315(1.153,1.450) | 1.045(0.935,1.178) | **<0.001** |
| Dorsum of hands | 1.165(0.945,1.413) | 1.025(0.950,1.120) | **0.027** |
| Anterior forearms | 1.250(1.040,1.513) | 1.080(0.950,1.158) | **<0.001** |
| Anterior upper arms | 0.860(0.753,1.098) | 0.865(0.765,0.940) | 0.532 |
| Forehead | 1.165(0.963,1.223) | 0.975(0.910,1.158) | 0.084 |
| Anterior chest wall | 1.525(1.208,1.673) | 1.230(1.180,1.428) | **0.047** |
| [Anterior](https://cn.bing.com/dict/search?q=anterior&FORM=BDVSP6&cc=cn) [abdominal](https://cn.bing.com/dict/search?q=abdominal&FORM=BDVSP6&cc=cn) [wa](https://cn.bing.com/dict/search?q=wall&FORM=BDVSP6&cc=cn)ll | 1.600(1.200,1.798) | 1.230(1.165,1.273) | **0.041** |
| Legs | 1.265(1.055,1.430) | 1.135(1.090,1.170) | **0.025** |
| Lateral lower legs | 1.020(0.960,1.198) | 1.020(0.870,1.108) | 0.052 |
| Dorsum of feet | 0.885(0.805,0.960) | 0.835(0.783,0.887) | **0.024** |
| **Skin stiffness** | **SSc (m/s)** | **HC (m/s)** | **P value** |
| Dorsum of hands | 4.415(3.833,5.190) | 2.940(2.683,3.885) | **<0.001** |
| Anterior forearms | 4.675(3.285,6.408) | 2.880(2.675,2.980) | **<0.001** |
| Anterior upper arms | 2.330(2.020,2.698) | 1.880(1.813,2.020) | **<0.001** |
| Anterior chest wall | 4.480(3.023,5.793) | 3.790(3.523,4.295) | 0.239 |
| [Anterior](https://cn.bing.com/dict/search?q=anterior&FORM=BDVSP6&cc=cn) [abdominal](https://cn.bing.com/dict/search?q=abdominal&FORM=BDVSP6&cc=cn) [wa](https://cn.bing.com/dict/search?q=wall&FORM=BDVSP6&cc=cn)ll | 3.970(2.728,4.733) | 3.005(2.723,3.620) | **0.026** |

**Supplementary Table S2** Comparison of skin thickness and stiffness between SSc patients with low and high disease activities.

| **Measured skin sites** | **Parameters** | | |
| --- | --- | --- | --- |
| **Skin thickness** | **HDAI-SSc (mm)** | **LDAI-SSc (mm)** | **P value** |
| Dorsum of middle fingers | 1.365(1.230,1.558) | 1.255(1.023,1.373) | **0.029** |
| Dorsum of hands | 1.265(0.985,1.480) | 1.025(0.833,1.378) | 0.135 |
| Anterior forearms | 1.330(1.005,1.703) | 1.210(1.073,1.325) | 0.276 |
| Anterior upper arms | 1.010(0.808,1.098) | 0.840(0.725,0.940) | **0.026** |
| Forehead | 1.195(1.078,1.283) | 0.990(0.930,1.190) | **0.026** |
| Anterior chest wall | 1.525(1.285,1.608) | 1.435(1.018,1.845) | 0.926 |
| [Anterior](https://cn.bing.com/dict/search?q=anterior&FORM=BDVSP6&cc=cn) [abdominal](https://cn.bing.com/dict/search?q=abdominal&FORM=BDVSP6&cc=cn) [wa](https://cn.bing.com/dict/search?q=wall&FORM=BDVSP6&cc=cn)ll | 1.600(1.200,1.798) | 1.605(1.098,2.150) | 0.806 |
| Legs | 1.265(1.138,1.843) | 1.215(0.898,1.405) | 0.184 |
| Lateral lower legs | 1.030(0.953,1.198) | 1.020(0.970,1.268) | 0.801 |
| Dorsum of feet | 0.925(0.853,1.028) | 0.875(0.735,0.905) | **0.031** |
| **Skin stiffness** | **HDAI-SSc (m/s)** | **LDAI-SSc (m/s)** | **P value** |
| Dorsum of hands | 4.800(4.188,6.260) | 3.845(3.238,4.408) | **<0.001** |
| Anterior forearms | 6.005(4.260,8.118) | 3.705(3.090,4.283) | **<0.001** |
| Anterior upper arms | 2.325(1.830,2.655) | 2.355(2.240,2.913) | 0.356 |
| Anterior chest wall | 5.390(3.893,6.100) | 3.215(2.020,4.580) | **0.038** |
| [Anterior](https://cn.bing.com/dict/search?q=anterior&FORM=BDVSP6&cc=cn) [abdominal](https://cn.bing.com/dict/search?q=abdominal&FORM=BDVSP6&cc=cn) [wa](https://cn.bing.com/dict/search?q=wall&FORM=BDVSP6&cc=cn)ll | 4.120(2.685,6.335) | 4.138(2.728,3.620) | 0.343 |

**Supplementary Table S3** ROC curve analysis for HFU to detect the skin thickness in SSc.

| **Measured skin sites** | **AUC** | **95%CI** | **P value** |
| --- | --- | --- | --- |
| Dorsum of middle fingers | 0.847 | 0.761-0.933 | **<0.001** |
| Dorsum of hands | 0.643 | 0.515-0.772 | **0.028** |
| Anterior forearms | 0.737 | 0.621-0.853 | **<0.001** |
| Anterior upper arms | 0.541 | 0.412-0.671 | 0.529 |
| Forehead | 0.660 | 0.490-0.830 | 0.083 |
| Anterior chest wall | 0.684 | 0.510-0.858 | **0.047** |
| [Anterior](https://cn.bing.com/dict/search?q=anterior&FORM=BDVSP6&cc=cn) [abdominal](https://cn.bing.com/dict/search?q=abdominal&FORM=BDVSP6&cc=cn) [wa](https://cn.bing.com/dict/search?q=wall&FORM=BDVSP6&cc=cn)ll | 0.689 | 0.508-0.869 | **0.041** |
| Legs | 0.645 | 0.514-0.775 | **0.026** |
| Lateral lower legs | 0.626 | 0.503-0.749 | 0.052 |
| Dorsum of feet | 0.646 | 0.522-0.770 | **0.025** |

**Supplementary Table S4** ROC curve analysis for SWE to detect the skin stiffness in SSc.

| **Measured skin sites** | **AUC** | **95%CI** | **P value** |
| --- | --- | --- | --- |
| Dorsum of hands | 0.879 | 0.807-0.951 | **<0.001** |
| Anterior forearms | 0.909 | 0.829-0.989 | **<0.001** |
| Anterior upper arms | 0.743 | 0.624-0.863 | **<0.001** |
| Anterior chest wall | 0.610 | 0.426-0.794 | 0.234 |
| [Anterior](https://cn.bing.com/dict/search?q=anterior&FORM=BDVSP6&cc=cn) [abdominal](https://cn.bing.com/dict/search?q=abdominal&FORM=BDVSP6&cc=cn) [wa](https://cn.bing.com/dict/search?q=wall&FORM=BDVSP6&cc=cn)ll | 0.705 | 0.530-0.880 | **0.027** |


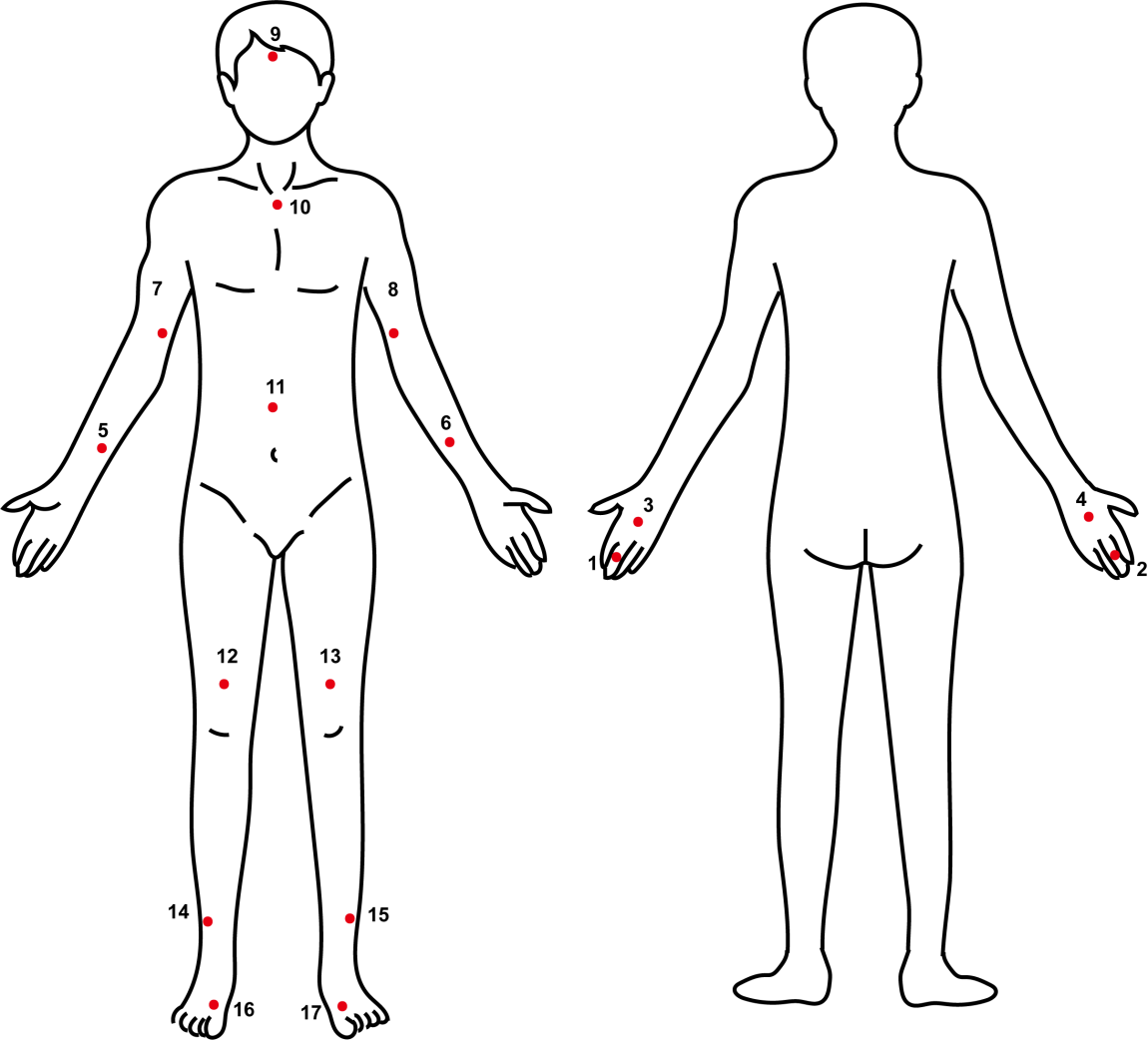


**Supplementary Figure S1**. Schema of 17 skin sites for skin ultrasound examination. (1-2) dorsum of middle fingers (proximal interphalangeal joint). (3-4) dorsum of hands (the metacarpal interspace of index and middle fingers, 2 cm proximal to the metacarpophalangeal joints). (5-6) anterior forearms (10 cm proximal to styloid process of ulna) (7-8) anterior upper arms (10 cm proximal to medial epicondyle). (9) forehead. (10) anterior chest wall (between jugular notch and sternal angle). (11) [anterior](https://cn.bing.com/dict/search?q=anterior&FORM=BDVSP6&cc=cn) [abdominal](https://cn.bing.com/dict/search?q=abdominal&FORM=BDVSP6&cc=cn) [wa](https://cn.bing.com/dict/search?q=wall&FORM=BDVSP6&cc=cn)ll (10 cm below xiphoid). (12-13) legs (10 cm proximal to patella). (14-15) lateral lower legs (10 cm proximal to lateral malleolus). (16-17) dorsum of feet (2 cm proximal to the 1^st^ and 2^nd^ metatarsophalangeal joints).
